# Stigma, discrimination and non-disclosure among young people living with HIV in Lagos, Nigeria

**DOI:** 10.11604/pamj.2022.41.106.27781

**Published:** 2022-02-07

**Authors:** Adedoyin Oyeyimika Ogunyemi, Fatimah Morenikeji Adubiaro, Esther Oluwakemi Oluwole, Esther Oluwatosin Somefun, Tope Olubodun

**Affiliations:** 1 Department of Community Health and Primary Care, College of Medicine, University of Lagos, Lagos, Nigeria,; 2 College of Medicine, University of Lagos, Lagos, Nigeria,; 3 United Nations Population Fund, Lagos, Nigeria,; 4 Department of Community Health, Lagos University Teaching Hospital, Lagos, Nigeria

**Keywords:** Discrimination, HIV, non-disclosure, reasons, stigma, young people

## Abstract

**Introduction:**

young people living with HIV (YPLH) constitute a significant population towards ending the AIDS epidemic. About half of YPLH are undiagnosed and one-third of new infections occurring among them. Stigma and discrimination remaina predominant enigma in the social response to HIV.

**Methods:**

this was a descriptive cross-sectional study among 124 YPLH aged 15-24 years selected by non-probability sampling from four antiretroviral centres targeted at young people across Lagos State. Ethical approval and informed consent were obtained. Data analysis was done using Epi info software version 7 and the level of significance was set at p<0.05.

**Results:**

the mean age of the participants was 19.4±3.2 years. Among the stigma variants, public stigma was the highest (48.4%), followed by anticipated stigma (20.2%), internalized stigma (14.5%), and enacted stigma (10.7%) while 7.3% of respondents experienced all forms. Thirty-seven percent of respondents had experienced one form of discrimination, with the most common form being ‘treated with hostility by strangers´ (14.5%). The disclosure level was 56.5%. The most predominant reasons for non-disclosure were fear of rejection by other people (57.3%). Diagnosis at an earlier age and living with a single parent were associated with lower disclosure levels (p‹0.001).

**Conclusion:**

overall stigma levels were found to be low, with differences in the individual stigma variants. The most common form of HIV-related discrimination reported in this study was being treated with hostility by strangers. Fear of rejection by other people was the main reason for non-disclosure among YPLH. The use of a multidisciplinary approach is needed to reduce the impact of stigma and discrimination among YPLH.

## Introduction

The burden of young people living with HIV (YPLH) has increased over the years. This is due in part to the effectiveness of the antiretroviral therapy (ART) and increased access to treatment improving the survival rate of infected children [1]. Recent estimates indicate the number of adolescents aged 130-19 years living with AIDS to be 160,000 and the national data also suggests that 40 percent of all stated new cases of HIV occur in young persons aged 15 to 24 [1]. Young people living with HIV constitute an important population in HIV prevention and management as their participation can easily be enlisted [2]. Adolescence and young adulthood is also a critical period of physical, cognitive and psychosocial development and as such are presumably more liable to the deleterious effects of stigma and discrimination [3]. Stigma and discrimination towards people living with HIV had since the outset of the AIDS epidemic been described as “the third phase of the epidemic” and was central to the global health challenge as the disease itself [4]. In spite of the advancements in HIV treatment as well as innovations of various means to educate the populace, HIV-related stigma still occupies the front seat in the social response to HIV/AIDS [5].

HIV-related stigma is “the prejudicial feelings, stereotypical perceptions, discriminatory behaviours and actions, or social devaluation of HIV infection, HIV/AIDS related illnesses, the activities associated with HIV-infection, and people with HIV” [6]. Discrimination involves exclusion of certain people from certain rights or benefits because they belong to a particular group that is regarded as “below others” and it also dates back as far as stigma in the HIV epidemic [7,8]. It differs from stigma in that discriminatory behaviours mostly come from other people rather than the infected person [7]. UNAID report from 50 countries indicate that an average of one in every eight people living with HIV is denied health services because of stigma and discrimination [9]. In the efforts to combat HIV, the disclosure of HIV serostatus has also attracted great attention as it has been seen to affect HIV transmission through decisions that hamper HIV prevention such as failure to disclose status to sexual partners [9,10]. Previous studies have shown that disclosure among YPLH has remained low due to fear of stigma and social rejection [11,12]. Statistics from the Nigeria AIDS Control Agency (NACA) stated the prevalence of HIV/AIDS at 4.2% for young people aged 15 to 24 [13]. This study examined stigma, discrimination, disclosure and related factors among YPLH in Lagos which is one of the “12+1 priority states for HIV control in a country with the second largest HIV epidemic globally.

## Methods

This study was a descriptive cross-sectional study carried out in four centres offering ART treatment for adolescents and young adults in Lagos State. The respondents were young people aged 15-24 years living with HIV attending ART clinics and currently on ART therapy. The sample size of 124 respondents was calculated using Cochran formula:


$$n=\frac{z^{2}pq}{d^{2}}$$

where n = the sample size, z = standard normal deviate at 95% confidence interval (CI) which is 1.96, p = prevalence of stigma and discrimination in the study population estimated as8% [14], q =1-p, d = precision level set at 5%. This was increased by 10% to account for non-response. All the four ART centres providing services to adolescents and young persons with HIV in Lagos State were selected. On every clinic day in each of the ART centre, all individuals who met the inclusion criteria were interviewed. Participants were thus enrolled consecutively until the sample size of 124 was reached.

The data was collected using semi-structured interviewer-administered questionnaire adapted from previous studies assessing stigma and discrimination [15,16]. This consisted of five sections: socio-demographic characteristics, reported health indices, assessment of stigma, assessment of discrimination, disclosure and disclosure challenges of respondents. The questionnaires were administered to the respondents by the researcher and trained research assistants at a convenient time during their clinic visit. The data was analysed using a computer statistical tool EPI Info version 7. Stigma was assessed with 15 questions using a 3-Likert scale: Never, Sometimes and most of the time. 'Never' was awarded a score of 0, 'Sometimes' a score of 1, and 'Most of the time', a score of 2. This gave a total obtainable score of 30. A score of 15 and above was taken as indicating stigma while a score below 15 was taken as the participant not stigmatized [17]. The individual variants of stigma were assessed using the mid-point of the total obtainable score in each variant. Discrimination was assessed using 7 questions. An answer of 'Yes' to any of the questions was taken to be indicative of discrimination. The results of the analysis were represented by the use of frequency tables and Chi-square test and the level of significance was set at p<0.05.

**Ethical approval:** it was obtained from the Health Research and Ethics Committee (HREC) of Lagos University Teaching Hospital (LUTH) with number ADM/DCST/HREC/APP/255and permission was gotten from the Lagos State Health service commission as well as from Heads or Medical Directors of the ART centres as applicable. Written informed consent was obtained from every respondent and strict confidentiality was maintained throughout in terms of participant´s identity and information provided.

## Results

The mean age of respondents was 19.4±3.2 years with about half of them (52.4%) being adolescents (15-19 years) and the rest of them being young adults (20-24 years). The majority of the respondents were female (64.5%) and the respondents currently attending school were more than half (53.2%). Also, 26.6% of the respondents live with single parents, 43.6% reside with both parents, 10.5% live with their spouse, 8.1% live alone, and 11.3% live with other relatives. About 1/3 of the respondents were diagnosed since birth (33.1%), and the mean age at diagnosis is 10±8.6 years. Less than half (43.6%) of the respondents assessed their health as very good while, 32.3% reported excellent and only 0.8% reported poor health status (Table 1). Stigma variants were assessed and public stigma was the highest (48.4%). Anticipated stigma was 20.2%, internalized stigma 14.5% and enacted stigma 10.7% (Table 2). In Table 3 the most common form of discrimination experienced by respondents is being treated with hostility by strangers (14.5%). Averagely, a tenth (11.3%) of the respondents have been avoided by people close to them, been rejected by a potential sexual or romantic partner, have been insulted or made fun of and have been treated poorly or made to feel inferior when receiving health care. The physically assaulted were 10.5% while 7.3% have had their personal properties damaged. This study reflected that 37.1% of the respondents have experienced one form of discrimination or the other within the past one year.

**Table 1:** socio-demographic characteristics of the respondents

Variable	Frequency (n=124)	Percentage (%)
**Age (years)**		
15-19	65	52.4
20-24	59	47.6
Mean age 19.4±3.2		
**Sex**		
Male	44	34.5
Female	80	64.5
**Currently in school**		
Yes	66	53.2
No	58	46.8
**Highest level of education**		
None	4	3.2
Primary	4	3.2
Secondary	72	58.1
Post-Secondary	44	35.5
**Resides with**		
Both parents	54	43.6
Single parents	33	26.6
Spouse	13	10.5
Alone	10	8.1
Others	14	11.3
**Age at diagnosis (years)**		
At Birth	41	33.0
1-5	7	5.7
6-10	15	12.1
11-15	15	12.1
16-20	29	23.4
21-24	17	13.7
**Mean age at diagnosis 10±8.6**		
**Reported health status**		
Excellent	40	32.2
Very Good	54	43.6
Good	25	20.2
Fair	4	3.2
Poor	1	0.8

**Table 2:** respondents experiencing stigma in the various stigma variants assessed (n=124)

Variable	Stigmatized (%)	Not stigmatized (%)
Anticipated stigma	25 (20.2)	99 (79.8)
Enacted stigma	13 (10.7)	111 (89.3)
Internalized stigma	18 (14.5)	106 (85.5)
Public stigma	60 (48.4)	64 (51.6)
Respondents with all stigma variant	9 (7.3%)	115 (92.7%)

**Table 3:** experiences of discrimination among respondents in the past year (n=124)

Variable	Yes (%)	No (%)	Don't know (%)
In the past 1 year: ignored, excluded, or avoided by close people	14 (11.3)	95 (76.6)	15 (12.1)
Rejected by a potential sexual or romantic partner	14 (11.3)	91 (73.4)	19 (15.3)
Treated with hostility or coldness by strangers	18 (14.5)	86 (69.3)	20 (16.1)
Insulted or made fun of	14 (11.3)	98 (79.0)	12 (9.7)
Personal property damaged or stolen	9 (7.3)	107 (86.3)	8 (6.5)
Physically assaulted or beaten up	13 (10.5)	105 (84.7)	6 (4.8)
Treated poorly or made to feel inferior when receiving health care	14 (11.3)	99 (79.8)	11 (8.9)
Has experienced at least one form of discrimination	46 (37.1)	78 (62.9)	0 (0.0)

In Table 4, over half of the respondents (59.7%) felt that they should be responsible for disclosing their HIV status while 29.0% of them also felt the information should be disclosed by the doctors and 9.7% felt it should be disclosed by their parents. About 27.4%, 16.9% and 14.5% of the respondents disclosed to their parents, sexual partners and siblings by themselves respectively. The most predominant reason for disclosure was expectation of care and emotional support (34.7%). Others were the need for prayer and company (19.4%), so that the informed person could get tested (16.9%), and the expectation of financial support (10.5%) from such persons. On the other hand, reasons for non-disclosure were fear of other people getting to know their status (57.3%), fear of being gossiped about (47.6%), fear of bad treatment from informed person (33.9%), fear of sadness upon disclosure (30.7%), and fear of accusation of promiscuity (25.8%). Other reasons for non-disclosure were feeling of entitlement to private life and belief that other people need not know their HIV status. Table 5 reflects the relationship between socio-demographic characteristics and having disclosed to at least one person. Young adults in the 20-24 age group (52.5%) were more likely to disclose their HIV status to someone else aside from their parents compared to the adolescents in the 15-19 age group (20.0%). Females (42.5%) and respondents who reside with their spouse (76.9%) or alone (70.0%) were more likely to disclose to others aside from their parents.

**Table 4:** factors affecting disclosure (n=124)

Variable	Yes (%)	No (%)
Respondents think the following people should be responsible for disclosure of their HIV status		
Self	74 (59.7)	50 (40.3)
Parents	12(9.7)	112 (90.3)
Other relatives	0(0.0)	124 (100.0)
Doctors	36(29.0)	88 (71.0)
Religious leaders	1(0.8)	123 (99.2)
**Persons respondents personally disclosed to**		
Parents	34 (27.4)	90 (72.6)
Siblings	18 (14.5)	106 (85.5)
Other relatives	4 (3.2)	120 (96.8)
Friends	2 (1.6)	122 (98.4)
Sexual partner	21 (16.9)	103 (83.1)
School teachers	1 (0.8)	123 (99.2)
Religious leaders	3 (2.4)	121 (97.6)
**Disclosed to**		
No one	9 (7.3)	115 (92.7)
Only parents	54 (43.6)	70 (56.5)
Other people aside from parents	44 (35.6)	80 (64.5)
Disclosure level	70 (56.5%)	54 (43.5%)
Reasons for disclosure	43 (34.7)	81 (65.3)
Expectation of care and encouragement	13 (10.5)	111(89.5)
Expectation of financial support	24 (19.4)	100 (80.6)
Expectation of prayer and company	24 (19.4)	100 (80.6)
To be able to live a normal life around them	21 (16.9)	103 (83.1)
Need for the informed person to also get tested	6 (4.8)	118 (95.2)
**Other reasons**		
Reasons for non-disclosure		
Fear of other people getting to know	71 (57.3)	53 (42.7)
Feeling of sadness upon disclosure	38 (30.7)	86 (69.3)
Fear of bad treatment from informed person	42 (33.9)	82 (66.1)
Fear of being accused of being promiscuous	32 (25.8)	92 (74.2)
Fear of being gossiped about	59 (47.6)	65 (52.4)
Other reasons	7 (5.7)	117 (94.3)

**Table 5:** association of socio-demographic characteristic and disclosure status

Sociodemographic characteristic	Disclosed (%) n=44	Not disclosed (%) n=80	χ2	p
**Age**				
15-19	13 (20.0)	52 (80.0)	14.3	*<0.001
20-24	31 (52.5)	28 (47.5)		
Age at diagnosis (years)				
<10	14 (23.3)	46 (76.7)	7.5	*0.006
10 and above	30 (46.9)	34 (53.1)		
**Sex**				
Male	10 (22.7)	34 (77.3)	4.8	*0.028
Female	34 (42.5)	46 (57.5)		
**Reside with**				
Both parents	15 (27.8)	39 (72.2)	8.5	*<0.001
Single parents	9 (27.3)	24 (72.7)		
Spouse	10 (76.9)	3 (23.8)		
Alone	7 (70.0)	3 (30.0)		
Others	3 (21.4)	11 (78.6)		

* Statistically significant association

## Discussion

Only a small proportion (7.3%) of the respondents was found to be having all forms of the stigma variants in this study. This is similar to a study conducted in Ido-Ekiti, Nigeria where 8% of the sample population were found to have a high level of stigmatization [14]. The low level of stigma can be attributed to Okoror *et al*. explanation of the correlation of stigma and ART treatment. They explained that due to the better physical appearance afforded People Living with HIV/AIDS (PLWHA) by the drugs, stigma levels expectedly reduced [18,19]. Furthermore, the low level of stigma among respondents in this study may be adduced to be an as a result of the comfort of residing with their parents or spouses and such stability may be a safety net. This level of stigma is in contrast to a study done among people living with HIV attending a general hospital in Island, Lagos [17]. In their study, general stigma level was 35%, this variation in total stigma levels is likely due to age difference in study population, self-dependence and personal responsibility as participants were aged between 15 and 64 years. Among the stigma variants assessed in this study, public stigma was found to be highest, followed by anticipated stigma, internalized stigma and enacted stigma. This trend is similar to that observed in a study among adolescents in South Africa, where enacted stigma was lower than anticipated stigma and internalized stigma [16]. Similarly, the study in the general hospital, Lagos reported that public stigma was the variant of stigma experienced by majority respondents [17]. Overall, 37.1% of respondents in this study had experienced at least one form of discrimination in the last one-year due to their HIV status. A lower figure was observed in an Ibadan study among youths [20]. Older studies in India (70.0%) [21] and France (33.3%) [22], United States (45.0%) [18] showed varying levels of discrimination among its respondents. The different levels of reported discrimination are probably due to variation in the study populations and level of awareness on HIV/AIDS at the time the research studies were conducted.

The most common form of HIV-related discrimination reported in this study was being treated with hostility by strangers. Others were, being ignored, excluded, or avoided, being rejected by a potential sexual or romantic partner, being insulted or made fun of and being treated poorly or made to feel inferior when receiving health care. A study among black men living with HIV in the United States most common forms of HIV-related discrimination were being rejected by potential sexual/romantic partners, being insulted or made fun of, and being ignored, excluded, or avoided by close others [18]. The study´s finding of being rejected by a potential romantic partner as the most common form of discrimination are in keeping with their adult status. In this study, the level of disclosure to at least one person was high, about 92.7%. A similarly high disclosure level (81.8%) [23] and (80.0%) [20] were reported in a study carried out among youth living with HIV in different studies in Ibadan and Niger Delta region of Nigeria (77.0%) [24]. This is not unexpected as parents are the primary caregivers of HIV infected adolescents and a significant number of the respondents were perinatally infected. However, in this study, when we assessed for disclosure to another person aside from respondent´s parents, disclosure level dropped to 56.5% for the obvious reason stated above. After disclosure to parents in over a quarter, it was followed by sexual partners (16.9%), siblings (14.5%) and other adult relatives. This finding is similar to that observed in a study among youths living with HIV in Ibadan, Nigeria [23]. The study reported that common people first informed included respondent´s mother, then spouse, father and sibling. Available literature most often indicates that disclosure to a sexual partner is challenging for adolescents and young persons because of fear of rejection or other forms of stigma and questions about how the virus was acquired [25]. However, higher values of disclosure to sexual partners were seen in previous studies in Ibadan (45.3%) [23], (66.3%) [20] and REACH in United States (47.5%) [26]. A different trend was observed in another Nigerian study, which found disclosure most prominent to pastors [24]. Followers tend to trust their religious leaders more for confidentiality, emotional healing and support. Another study on disclosure of serostatus among youths showed a predisposition to disclose to friends (89%) and family members (87%) [27]. The reason for the higher disclosure to others aside parents in other studies may be accounted for by the older ages of the respondents.

The most predominant reason for disclosure in this study was expectation of care and emotional support (34.7%) followed by need for prayer and company (19.4%), need for the informed person to get tested (16.9%), and expectation of financial support (10.5%). Similar findings were reported in a study conducted to assess serostatus disclosure in a resource limited setting in the Niger delta area of Nigeria, as disclosure was reported to be mostly associated with expectation of spiritual support, expectation of economic support, expectation of social support and expectation of emotional support [24], This suggests that the choice of who to disclose to depend on who respondents find likely to give them support. For non-disclosure in this study, predominant reasons included fear of the following; others getting to know, being gossiped about, bad treatment from the informed person, sadness upon disclosure, and fear of accusation of promiscuity. Fear of stigmatization, victimization, accusation of infidelity and spreading news of serostatus were reported as major reasons for non-disclosure in a previous study [19]. Reasons such as these were also given for non-disclosure among respondents in Ogun State [28].

In this study, the older age group of 20-24 disclosed their HIV status to someone else aside from their parents compared to the adolescents in the 15-19 age group and this finding was statistically significant (p<0.001). This was similar to the finding among people living with HIV/AIDS in Ogun State, Nigeria [28] reported that disclosure was significantly associated with age. This could be due to emotional maturity to handle the consequences of disclosure and independence on the part of the older age group. However, some previous studies found no association between age and disclosure [14,23]. Also, disclosure was also found to be associated with age of disease diagnosis, as respondent diagnosed 10 years and above had a higher disclosure rate compared to those diagnosed earlier. A study on self-disclosure of serostatus by youth who are HIV-positive reported similar result [25], whereas another study [27] found no association. More females than males were likely to disclose their HIV status to others aside from their parents [24,29]. This is similar to the study in 3 African countries that found that more men than women were significantly less likely to report disclosing their HIV status to their sex partner(s) [30]. However, other researchers found no gender differences in HIV disclosure [31-33]. Other contrasting indigenous studies to our findings were reported in Oyo and Ogun States [23]. The diversity in research findings on the association between gender and disclosure of HIV status might be attributed to the fact that other factors such as a perception of the aftermath of disclosure could have a greater influence on disclosure than gender. This is consistent with the consequences of disclosure theory [34]. Also, respondents who reside with their spouses (76.9%) or alone (70.0%) were more likely to disclose to others apart from their parents. The studies in Ibadan reflect corresponding results [20,23]. This also can be explained by maturity and independence on the part of this population of respondents.

## Conclusion

Overall stigma levels were found to be low, with differences in the individual stigma variants. The most common form of HIV-related discrimination reported in this study was being treated with hostility by strangers. Disclosure to a significant other was high; however, disclosure to other people aside from respondents´ parents was lower. Continuous sensitization on benefits of disclosure among of young people on the benefit of disclosure should be encouraged. Interventions directed at adolescents should be made to include their families, as family support has been shown to affect stigmatization and discrimination in this study. Mental Health and Psychosocial Support (MHPSS) should be commenced upon diagnosis to young HIV positive people living alone and with single parents to attenuate their psychological distress and reduce their susceptibility to stigma and discrimination.

### What is known about this topic

- Young people living with HIV experience both stigma and discrimination;
- More females than males experience discrimination;
- Disclosure of HIV status by YPLH were mostly to their parents and spouses.

### What this study adds

- The variant of stigma experienced by YPLH found public stigma to be highest and enacted stigma as the least;
- There was a notable drop in proportion of disclosure levels to another person aside from respondent´s parents;
- The most predominant reason for disclosure, in this study, was expectation of care and emotional support.

## References

[1] Idele P, Gillespie A, Porth T, Suzuki C, Mahy M, Kasedde S (2014). Epidemiology of HIV and AIDS among adolescents: current status, inequities, and data gaps. J Acquir Immune Defic Syndr.

[2] Joint United Nations Program on HIV and AIDS (2016). Ending the AIDS epidemic for adolescents with adolescents.

[3] Woollet N, Black V, Cluver L, Brahmbhatt H (2017). Reticence in disclosure of HIV infection and reasons for bereavement: impact on perinatally infected adolescents´ mental health and understanding of HIV treatment and prevention in Johannesburg, South Africa. Afr J AIDS Res.

[4] Mann JM (1988). Statement at an informal briefing on AIDS to the 42^nd^ session of the united nations general assembly. J R Stat Soc Ser A Stat Soc.

[5] Chambers LA, Rueda S, Baker DN, Wilson MG, Deutsch R, Raeifar E (2015). Stigma, HIV and health: a qualitative synthesis. BMC Public Health.

[6] Vorasane S, Jimba M, Kikuchi K, Yasuoka J, Nanishi K, Durham J (2017). An investigation of stigmatizing attitudes towards people living with HIV/AIDS by doctors and nurses in Vientiane, Lao PDR. BMC Health Serv Res.

[7] Nöstlinger C, Castro DR, Platteau T, Dias S, Gall JL (2014). HIV-related discrimination in European health care settings. AIDS Patient Care STDS.

[8] Skinner D, Mfecane S (2004). Stigma, discrimination and the implications for people living with HIV/AIDS in South Africa. J Soc Asp HIV/AIDs.

[9] Joint United Nations Programme on HIV/AIDs (2017). Make some noise for zero discrimination.

[10] Siu GE, Bakeera-Kitakab S, Kennedy CE, Dhabangia A, Kambugud A (2012). HIV serostatus disclosure and lived experiences of adolescents at the transition clinic of the infectious diseases clinic in Kampala, Uganda: a qualitative study. AIDS Care.

[11] Michaud P, Suris J, Thomas RP, Kahlert C, Rudin C, Cheseaux J (2009). To say or not to say: a qualitative study on the disclosure of their condition by human immunodeficiency virus-positive adolescents. J Adolesc Health.

[12] Birungi H, Obare F, Mugisha JF, Evelia H, Nyombi J (2009). Preventive service needs of young people perinatally infected with HIV in Uganda. AIDS Care.

[13] National Agency for the Control of AIDS (NACA) Global, AIDS (2014). Response country progress report: Nigeria GARPR.

[14] Omosanya OE, Elegbede OT, Agboola SM, Isinkaye AO, Omopariola OA (2014). Effects of stigmatization/discrimination on antiretroviral therapy adherence among HIV-infected patients in a rural tertiary medical center in Nigeria. J Int Assoc Provid AIDS Care JIAPAC.

[15] Bogart LM, Wagner GJ, Galvan FH, Landrine H, Klein DJ, Sticklor LA (2011). Perceived discrimination and mental health symptoms among black men with HIV. Cult Divers Ethn Minor Psychol.

[16] Pantelic M, Boyes M, Cluver L, Meinck F (2017). HIV, violence, blame and shame: pathways of risk to internalized HIV stigma among South African adolescents living with HIV. J Int AIDS Soc.

[17] Sekoni AO, Obidike OR, Balogun MR (2012). Stigma, medication adherence and coping mechanism among people living with HIV attending General Hospital, Lagos Island, Nigeria. Afr J Prim Health Care Fam Med.

[18] Stephenson R (2009). Community factors shaping HIV-related stigma among young people in three African countries. AIDS Care.

[19] Okoror TA, Falade CO, Olorunlana A, Walker EM, Okareh OT (2013). Exploring the cultural context of HIV stigma on antiretroviral therapy adherence among people living with HIV/AIDS in southwest Nigeria. AIDS Patient Care STDS.

[20] Adesola O, Owoaje E (2012). Experiences of discrimination among youth with HIV/AIDS in Ibadan, Nigeria. J Public Health Afr.

[21] ILO Decent work team for South Asia and ILO office for India (2003). Economic impact of HIV/AIDS and their families.

[22] Lert F, Obaia Y, Dray-Spira R (2010). Study of the social situation of persons living with HIV/AIDS and the responsiveness of the health care system and social services.

[23] Olumide A, Owoaje E (2018). Patterns and predictors of disclosure of HIV positive status among youth living with HIV in Ibadan, Nigeria. Int J Adolesc Med Health.

[24] Akani CI, Erhabor O (2006). Rate, pattern and barriers of HIV serostatus disclosure in a resource-limited setting in the Niger delta of Nigeria. Trop Doct.

[25] Thoth CA, Tucker C, Leahy M, Stewart SM (2014). Self-disclosure of serostatus by youth who are HIV-positive: are view. J Behav Med.

[26] D´Angelo LJ, Abdalian SE, Sarr M, Hoffman N, Belzer M (2001). Disclosure of serostatus by HIV infected youth: the experience of the REACH study, Reaching for Excellence in Adolescent Care and Health. J Adolesc Health.

[27] Lee M, Rotheram-Borus MJ, O´hara P (1999). Disclosure of serostatus among youth living with HIV. AIDS Behav.

[28] Amoran OE (2012). Predictors of disclosure of serostatus to sexual partners among people living with HIV/AIDS in Ogun State, Nigeria. Niger J Clin Pract.

[29] Dempsey AG, MacDonell KE, Naar-King S, Lau CY (2012). Patterns of disclosure among youth who are HIV-positive: a multi-site study. J Adoles Health.

[30] Bachanas P, Medley A, Pals S, Kidder D, Antelman G, Benech I (2013). Disclosure, knowledge of partner status, and condom use among HIV-positive patients attending clinical care in Tanzania, Kenya, and Namibia. AIDS Patient Care STDs.

[31] Deribe K, Woldemichael K, Wondafrash M, Haile A, Amberbir A (2008). Disclosure experience and associated factors among HIV positive men and women clinical service users in southwest Ethiopia. BMC Public Health.

[32] Wiener LS, Battles HB (2006). Untangling the web: a close look at diagnosis disclosure among HIV-infected adolescents. J Adolesc Health.

[33] Nöstlinger C, Bakeera-Kitaka S, Buyze J, Loos J, Buvé A (2015). Factors influencing social self-disclosure among adolescents living with HIV in Eastern Africa. AIDS Care.

[34] Serovich JM (2001). A test of two HIV disclosure theories. AIDS Educ Prev.

